# Overexpression of lncRNA H19 changes basic characteristics and affects immune response of bovine mammary epithelial cells

**DOI:** 10.7717/peerj.6715

**Published:** 2019-04-05

**Authors:** Xuezhong Li, Hao Wang, Yanfen Zhang, Jinjing Zhang, Shaopei Qi, Yong Zhang, Ming-Qing Gao

**Affiliations:** 1 College of Veterinary Medicine, Northwest A&F University, Yangling, China; 2 Northwest A&F University hospital, Northwest A&F University, Yangling, China

**Keywords:** Bovine mastitis, Epithelial cells, Long non-coding RNA H19, Lipopolysaccharide, Immnue response

## Abstract

The function of long non-coding RNA H19 (H19) on cell proliferation has been observed in various cell types, and the increased expression of H19 was also found in the lipopolysaccharide (LPS)-induced inflammatory bovine mammary epithelial cells (MAC-T). However, the roles of H19 in the inflammatory response and physiological functions of bovine mammary epithelial cell are not clear. In the present study, we found that overexpression of H19 in MAC-T cells significantly promoted cell proliferation, increased the protein and mRNA level of β-casein, and enhanced the expression of tight junction (TJ)-related proteins while inhibited *staphylococcus aureus* adhesion to cells. In addition, results demonstrated that overexpression of H19 affected the LPS-induced immune response of MAC-T cells by promoting expressions of inflammatory factors, including TNF-α, IL-6, CXCL2 and CCL5, and activating the NF-κB signal pathway. Our findings indicate that H19 is likely to play an important role in maintaining normal functions and regulating immune response of bovine mammary epithelial cells.

## Introduction

Bovine mastitis is one of the most common diseases of dairy cow. It is usually caused by the abnormal changes of physiological status, mammary damage and environmental pathogenic microorganisms. Mammary epithelium not only stands as a barrier of mammary glands of dairy cow from the environment, but also functions in the process of milk synthesis and secretion. Recent studies demonstrate that mammary epithelial cells mediate the immune response by regulating the expression of immune factors once pathogenic microorganisms invade mammary glands, contributing to the development of inflammation (Gunther et al., 2012). The classical inflammation-related pathways are activated in the inflammatory process of mammary epithelial cells induced by pathogens or their virulence components, such as the NF-κB pathway (He et al., 2017b), the MAPK pathway (Wang et al., 2016), the TLRs pathway (De Schepper et al., 2008) and the JAK-STAT pathway (Usman et al., 2014). The inflammation usually causes dysfunction of mammary glands, as Kobayashi confirmed that injection of LPS, the major structural elements of the *Escherichia coli* (*E. coli*) cell membrane, into the mouse mammary gland could cause the leak of β-casein from mammary alveolar to interstitial, coincided with the damage of tight junction (TJ) structure of mammary epithelial cells in inflammatory conditions (Kobayashi et al., 2013). We have reported that TJ plays a pivotal role in maintaining the integrity of blood-milk barrier in bovine mammary glands under an inflammatory condition in previous publication (Xu et al., 2018).

Long non-coding RNA (lncRNA) is a class of RNAs with more than 200 nucleotides in length and poor capability to encode protein. LncRNA also can function by mediating DNA methylation, histone modification, chromatin remodeling, mRNA degradation and protein modification (Gong & Maquat, 2011; Mercer, Dinger & Mattick, 2009; Pandey & Kanduri, 2011; Tani et al., 2012; Tsai et al., 2010). Numerous evidences suggest that lncRNAs play key roles in the regulation of expressions of specific genes in pathogens-induced acute and chronic inflammation (Carpenter & Fitzgerald, 2015). As the first identified lncRNA, it has been reported that H19 may play a dual role of oncogene and tumor suppressor gene (Matouk et al., 2007; Matouk et al., 2014; Yoshimizu et al., 2008). H19 promotes breast cancer cell proliferation through positive control by E2F1 (Berteaux et al., 2005). Furthermore, recent findings indicate that H19 promotes neuroinflammation by driving HDAC1-dependent M1 microglial polarization (Wang et al., 2017). These studies demonstrate that H19 is widely involved in physiological and pathological processes including multiple inflammations. Our previous work showed that H19 was significantly upregulated in bovine mammary epithelial cells under inflammatory condition and in bovine mastitic mammary tissue (Yang et al., 2017). However, the roles of H19 in the normal function maintenance and inflammatory response of bovine mammary epithelial cells remain unclear.

In this study, we explored the functions of H19 in cell proliferation, apoptosis, β-casein gene expression and protein secretion, TJ-related protein expression and bacterial adhesion of mammary epithelial cell by overexpressing H19.

## Materials & Methods

### Cell culture and treatment

The stablely over-expressing H19 MAC-T cell were established in our previous work (Yang et al., 2017). Both MAC-T cells and the H19-overexpressed MAC-T cells were routinely maintained at 37 °C in an incubator with 5% CO_2_ in sterile cell culture dishes containing DMEM/F12 medium (Gibco BRL, Grand island, NY, USA) mixed with 10% fetal bovine serum (FBS), 100 IU/mL penicillin and 100 µg/mL streptomycin. The inflammatory cell model is established with LPS (Sigma-Aldrich, St Louis, MO, USA) at 10 ng/µL for 3 h according to the recommended conditions in our previous publication (Zhang et al., 2016).

### RNA extraction and real-time quantitative PCR (RT-qPCR)

Total RNA from the LPS-unstimulated and -stimulated cells was obtained by using ice-cold TriZol reagent (TransGene, Shanghai, China) following the manufacturer’s instructions. The concentration of the total RNA is determined by spectrophotometry (BioTek, Winooski, VT) at an optical density of 260 nm. The quality of the RNA is monitored by A260/A280 ratio (1.8∼2.0). Then 2 µg mRNA is reverse-transcribed into cDNA with TransScript II First-Strand cDNA Synthesis SuperMix (TransGene, Shanghai, China). RT-qPCR was carried out on ABI Step One Software System (ABI, Foster City, CA) using HotStart SYBR Green qPCR Master Mix (USB, Cleveland, OH). GAPDH was used as a reference gene. All PCR primers were synthesized by Sangon Biotech (Sangon, Shanghai, China).

### Western blotting analysis

Total proteins were extracted from cells that had grown up to around 80% confluence using PRO-PREP Protein Extraction Solution (iNtRON Biotechnology, Inc. Gyeonggi-do, Korea). The protein concentration of cell lysate was measured by Bradford protein assay kit (TransGene, Beijing, China), and 30 µg of every protein sample was loaded and separated by SDS-PAGE. Subsequently, the separated proteins were electrotransferred onto polyvinylidene fluoride membranes (Millipore, Bedford, MA, USA). After being blocked with 10% nonfat milk, the membranes were subsequently incubated overnight at 4 °C with the following antibodies: Zonula occludens 1 (ZO-1, 1:1,000 diluted, Bioss, Beijing, China), Occludin (1:1000 diluted; Bioss), Claudin-1 (1:1,000 diluted, Bioss), P65 (1:500 diluted; Santa Cruz, Dallas, TX, USA), P-P65 (1:500 diluted; Bioss), Histone H3 (1:1,000 diluted; Beyotime, Shanghai, China), GAPDH (1:1,000 diluted; TransGen). Then the membranes were washed with PBS for three times and incubated with HRP-conjugated secondary antibodies (1:2,000 diluted; Beyotime). The positive bands were visualized by using enhanced chemiluminescence (Beyotime). Image J software with default settings was employed to quantify the grayscale of the western blots.

### ELISA

The protein concentration of TNF-α, IL-6, CCL2, and CXCL5 secreted in medium by cells were measured by ELISA kits. H19-overexpresed MAC-T cells were grown to 80% confluence in 60-mm culture dishes and then treated with LPS, while the cells treated with no stimulation was used as control. After treatment, the medium was replaced by the fresh culture with 10% FBS and antibiotics, then culture for another 24 h. Finally, the media was collected and cells debris were removed by centrifuge. TNF-α, IL-6, CCL2, CXCL5 and β-casein secreted in the medium was detected by corresponding ELISA kits (Huzhen Biological Technology, Shanghai, China) following the manufacturer’s protocol. MAC-T cells transduced with empty vector were uses as a control.

### Cell proliferation assay

Cell viability was assessed by a cell counting kit-8 (CCK-8) (Beyotime). In brief, cells were seeded into 96-well plate at a density of 2,000 cells/well. Then after cells attachment, cell viability was detected in three days in accordance with the manufacturer’s instructions. The absorbance of samples in triplicate wells were measured at 450 nm wavelength (OD450) using a microplate reader (BioTek), and the cell numbers were calculated based on a standard curve obtained under the same experimental conditions. The MAC-T cells transfected with the empty vector was used as a control.

### Cell apoptosis Assay

Cells apoptosis were analyzed by flow cytometry. Cells were routinely collected and resuspended in binding buffer and treated with the buffer dissolving Annexin V-phycoerythrin (PE) and 7-AAD for 15 min at room temperature in the dark. Then the apoptotic rate was detected and analyzed by flow cytometry.

### Bacterial adhesion analysis

Bacterial adhesion assays were based on a protocol described in a previous publication with slight modifications (Almeida et al., 1996). Cells were seeded in 24-well culture plates at 1.5 × 10^5^ cells/well and cultured for 24 h at 37 °C with 5% CO_2_ to reach confluence. The confluent cells were washed with PBS, then inoculated in 200 µL of invading medium (without antibiotic growth medium) containing about 10^7^ *S. aureus* for 1 h at 37 °C. Next, cells were washed three times with PBS, and routinely collected and resuspended in 1 mL sterile deionized water. The cell suspension was serially10-fold diluted and 10 µL of dilutions were plated on LB agar in triplicate. The numbers of adhesive *staphylococcus aureus* (*S. aureus*) per ml were determined by standard colony counting methodology. The experiments were repeated three times, and bacterial adhesion rate was calculated as follows: A_0_/A_n_, where A_0_ and A_n_ respectively represent the number of adhesive *S. aureus* and the total number of bacteria added per well.

### Luciferase assay

Cells were cultured with culture medium in 6-well plates. At 70∼80% confluence, cells were routinely collected and transferred into a 4-mm cuvette with transfer buffer. Cells were co-transfected with 5 µg of vector containing a responsive element to NF-κB driving the expression of firefly luciferase (pGL4.32 Luc2P-NF-κB®, Promega, Madison, WI) and 1 µg of renilla-luciferase construct (pRL-TK®, Promega) by electroporation at 510 V for one pulse. After transfecting for 12 h, the MAC-T cells were treated with 10 ng/µL LPS for 3 h at 37 °C in a humidified incubator. The untreated cells were used as control. Then cell culture supernatants were replaced with the fresh culture medium, and the cells were cultured for another 24 h. Finally, cells were lysed and luciferase activities were assessed by a Dual-Luciferase Reporter analytical instrument (Promega, Madison, WI, USA).

### Statistical analysis

The data were expressed as means ± standard deviation (SD). Every experiment had been repeated for at least three times. All statistical analyses were performed using ANOVA with the Bonferroni post hoc test (SPSS 11.5; IBM Corporation, Armonk, NY, USA). *P* < 0.05 was considered statistically significant.

## Results

### H19 promoted MAC-T cell growth

**Figure 1:**
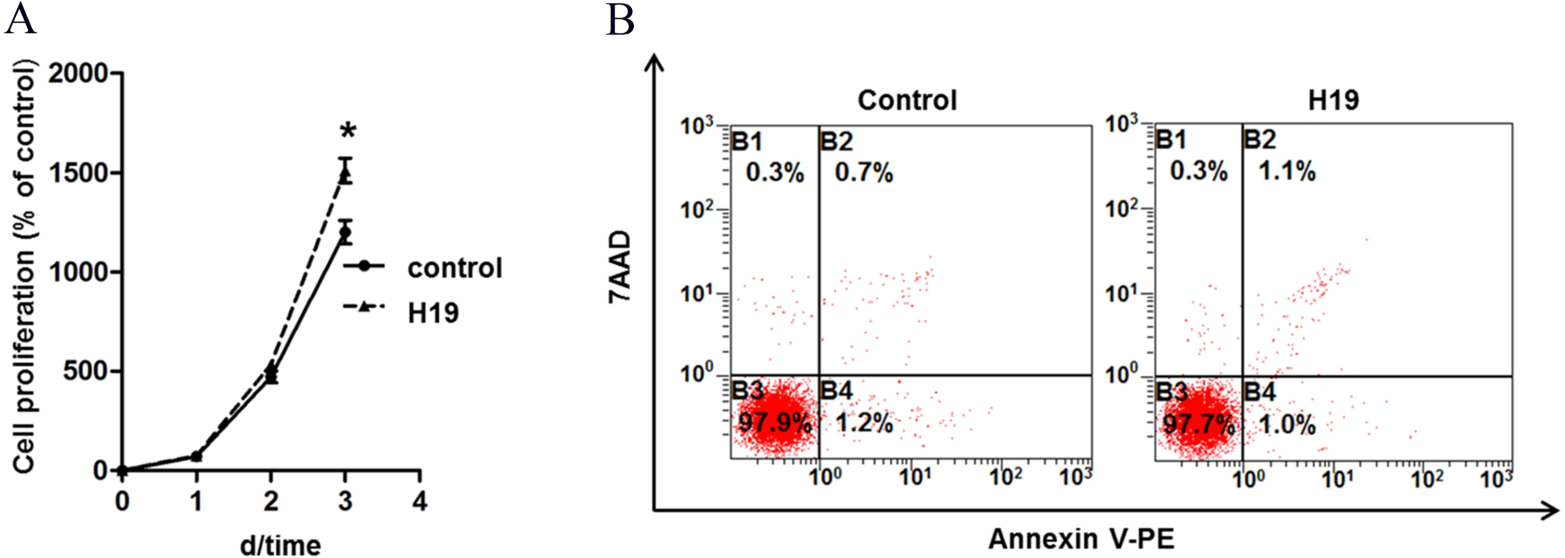
Overexpression of H19 promoted MAC-T cell proliferation but showed no effect on cell apoptosis. (A) Overexpression of H19 promoted the proliferation of MAC-T cells detected by CCK-8. *p < 0.05 vs. Control. (B) Overexpression of H19 did not induce MAC-T cell apoptosis. 7AAD was used to stain the cell nucleus of all non-viable cells, and phycoerythrin (PE)-labeled Annexin V bound the cytomembrane when cells were experiencing the early stage of apoptosis. All data were collected from three independently repeated experiments. The empty vector-transfected cells were used as Control.

To explore the role of H19 on MAC-T cells growth, we analyzed the proliferation and apoptosis of MAC-T cells by overexpressing H19. Results showed that H19 overexpression significantly promoted MAC-T cell proliferation (Fig. 1A) at the 3rd day after cell seeding, but we found no significant difference on cell apoptotic rates between two groups by using flow cytometry (Fig. 1B).

### H19 enhanced β-casein gene expression and protein secretion of MAC-T cells

**Figure 2:**
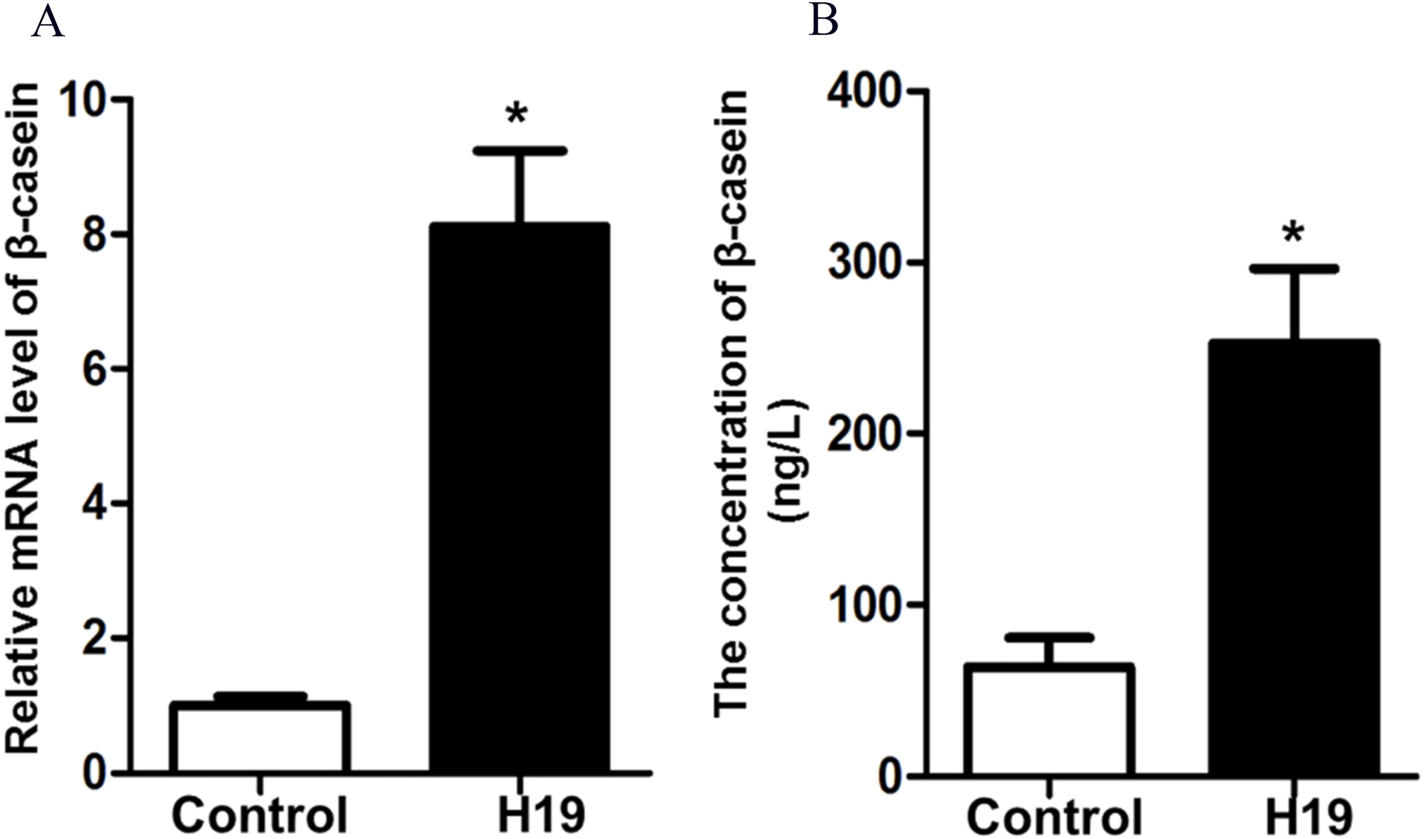
Overexpression of H19 increased β-casein gene and protein expression. (A) β-casein gene expression in H19-overexpressed MAC-T cells was analyzed by RT-qPCR. GAPDH was used as an internal control. (B) β-casein protein secretion in the medium was measured by using an ELISA kit. *p < 0.05 vs. Control.

To investigate the effects of H19 overexpression on β-casein gene expression and protein secretion of MAC-T cells, we detected β-casein mRNA expression and protein secretion by RT-qPCR and ELISA, respectively. Results showed that overexpression of H19 was able to significantly enhance mRNA expression (Fig. 2A) and protein secretion (Fig. 2B) of β-casein in MAC-T cells compared to those cells transfected with an empty vector.

### H19 changed the expression of TJ proteins of MAC-T cells

**Figure 3:**
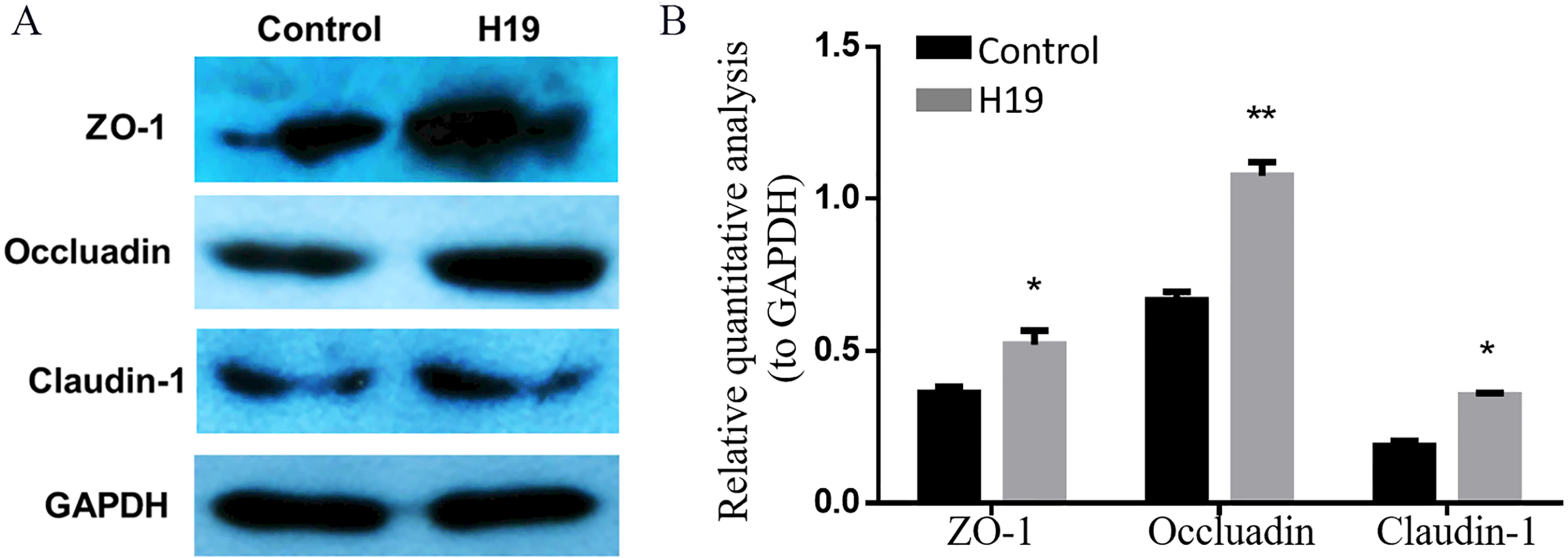
Overexpression of H19 upregulated the TJ-related proteins expression of MAC-T cells. (A) The expressions of TJ-related proteins of ZO-1, Occluadin, and Claudin-1 in H19-overexpressed MAC-T cells were analyzed by western blotting. The cells transfected with an empty vector were used as Control. (B) The quantitative analysis of each protein was relative to GAPDH. *p < 0.05 vs. Control.

To explore whether H19 is involved in maintaining the integrity of TJ of epithelial cells, we examined the expressions of several major proteins related to cell TJ, including ZO-1, Occludin and Claudin-1, by using western blotting. As shown in Figs. 3A and 3B, the expression levels of all three examined protein were higher in MAC-T cells with H19 overexpression compared to those with empty vector transfection.

### H19 inhibited *S. aureus* adhesion to MAC-T cells

**Figure 4:**
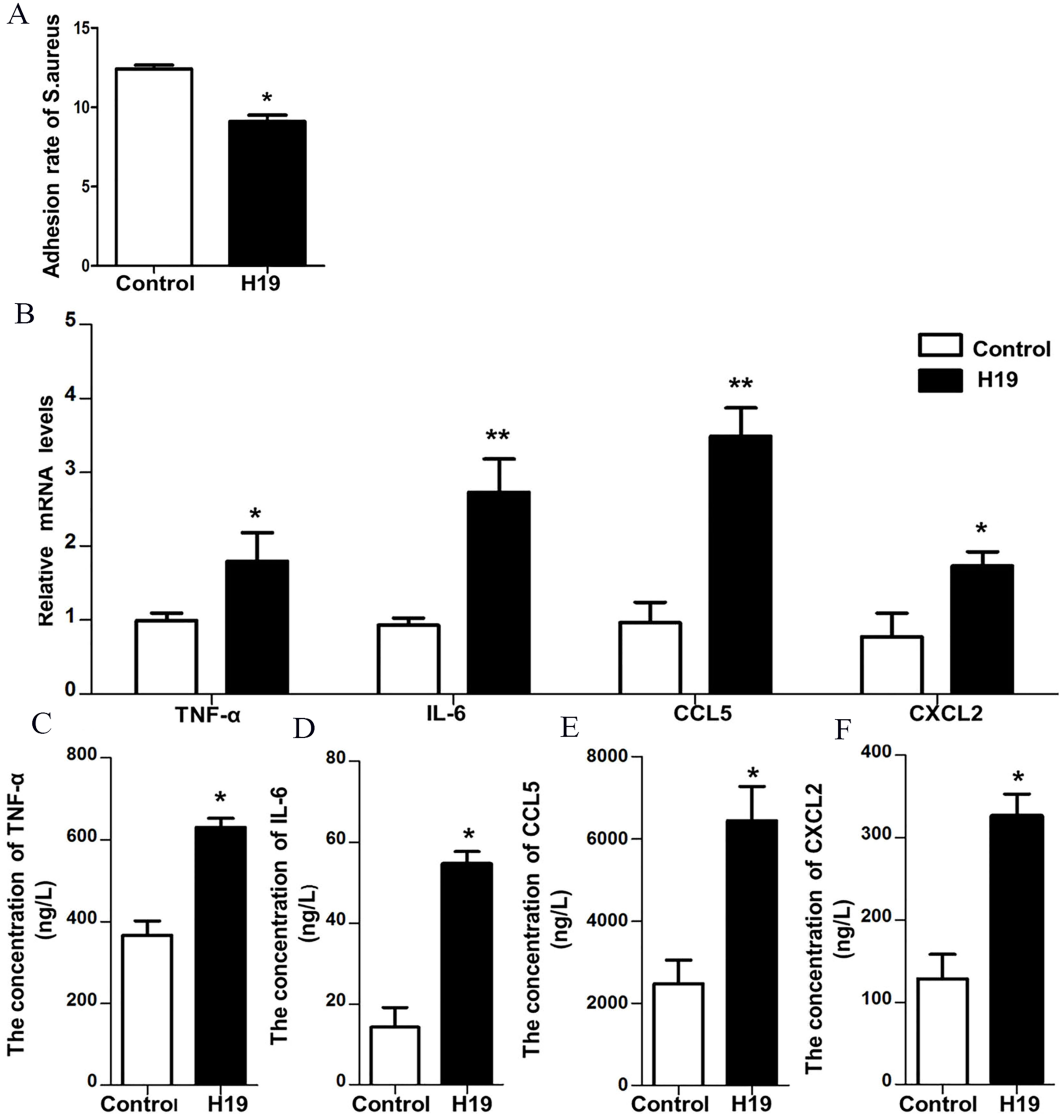
H19 inhibited Staphylococcus aureus adhesion to MAC-T cells and promoted expressions of inflammatory factors induced by LPS. (A) The effect of H19 on the adhesion of Staphylococcus aureus to MAC-T cells is evaluated by measuring the bacterial adhesion rate. Statistical analysis of the bacterial adhesion rate of Control and H19-overexpressed group was presented. The empty vector-transfected cells were used as Control. *p < 0.01 vs. Control. (B) Gene expression levels of TNF-α, IL-6, CCL5 and CXCL2 in H19-overexpressed MAC-T cells stimulated by LPS were determined by RT-qPCR. Empty vector-transfected cells were used as Control. *p < 0.05, **P < 0.01 vs. Control. Protein levels of TNF-α (C), IL-6 (D), CCL5 (E) and CXCL2 (F) secreted by H19-overexpressed MAC-T cells were measured with corresponding ELISA kits. Empty vector-transfected cells were used as Control. *p < 0.01 vs. Control.

To verify the role of H19 in the process of *S. aureus* adhesion to epithelial cells, a bacterial adhesion assay was performed. As shown in Fig. 4A, the adhesion rate of *S. aureus* was lower in MAC-T cells overexpressing H19 than those cells transfected with empty vector, indicating that H19 overexpression inhibited *S. aureus* adhesion into MAC-T cells.

### H19 was involved in LPS-induced inflammatory response of MAC-T cells through the NF-κB pathway

Our previous study suggested that LPS could trigger a strong inflammatory response of bovine mammary epithelial cells by introducing significant upregulation of inflammatory factors, including TNF-α, IL-6, CXCL2 and CCL-5 (He et al., 2017a; Yang et al., 2017; Zhang et al., 2018). To investigate whether H19 is involved in LPS-induced inflammatory response of MAC-T cells, the mRNA expression and protein secretion of these inflammatory cytokines and the activation of the NF-κB pathway were examined in the MAC-T cells. Results showed that overexpression of H19 upregulated both mRNA expression (Fig. 4B) and protein secretion levels (Figs. 4C–4F) of TNF-α, IL-6, CXCL2, and CCL5, suggesting that H19 promoted LPS-induced inflammatory responses in MAC-T cells.

**Figure 5:**
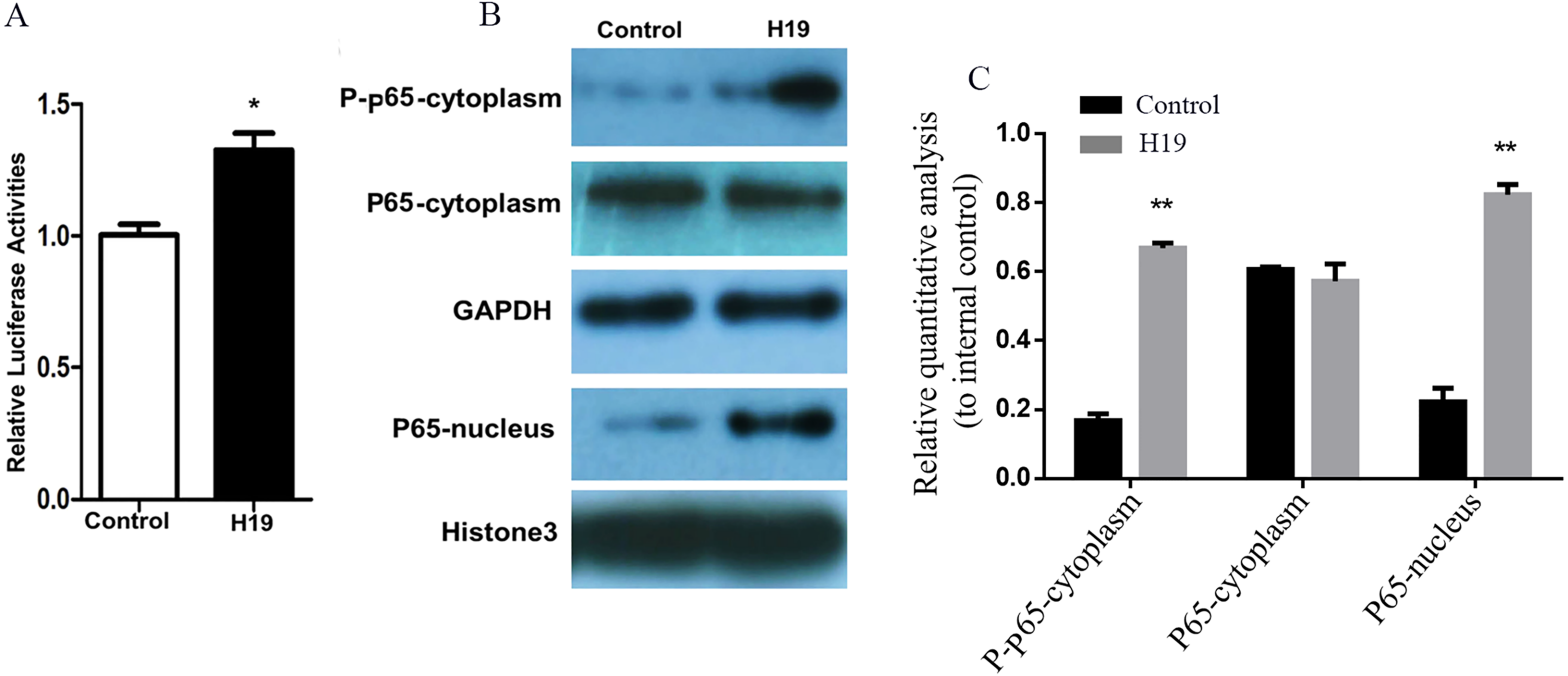
Overexpression of H19 in MAC-T cells intensified the activation of the NF-κB signal pathway. (A) NF-κB relative activity was quantified by transfecting the NF-κB reporter and using a luciferase assay in H19-overexpressed MAC-T cells stimulated by LPS. (B) The protein expressions of p-p65 and p65 in cells stimulated by LPS were analyzed by western blotting. The empty vector-transfected cells were used as Control. GAPDH and Histone H3 were respectively used as internal control for cytoplasm and nuclear proteins. The quantitative analysis of each protein was relative to internal control. *p < 0.05 vs. Control.

A gene reporter system of the NF-κB signaling pathway was used to detect the activation of this pathway, and results showed that the relative fluorescence of MAC-T cells overexpressing H19 was higher compared to that of control group (Fig. 5A). In addition, the expressions of key molecules in the NF-κB signaling pathway were detected by western blotting, and it was observed that the phosphorylated p65 and p65 entering into nuclear of MAC-T cell significantly increased after H19 overexpression (Figs. 5B and 5C).

## Discussion

As a costly disease, bovine mastitis always threatens the development of dairy industry. H19, a maternally expressed imprinted gene that is abundant in embryonic tissues but is strongly repressed after birth (Dey, Pfeifer & Dutta, 2014), is well studied on epithelial to mesenchymal transition (EMT) and mesenchymal to epithelial transition (MET) of various tumors, and our previous publication demonstrated that H19 was participated in the process of mastitis (Yang et al., 2017). In this study, we have evaluated the biological roles of H19 on cell growth and β-casein secretion of mammary epithelial cells by overexpressing H19. Consistent with the findings in previous publications (Liu et al., 2016; Yang et al., 2012; Yang et al., 2017), the promotion effects of H19 on proliferation of several types of cells were evidenced. We here confirmed that H19 overexpression promoted the cell growth but had no effect on apoptosis rate of the MAC-T cell. Correspondingly, increased β-casein secretion was observed because of its rapid proliferation caused by H19 overexpression.

Mastitis is characterized by decreased milk production and an influx of somatic cells into mammary alveolus due to the destruction of the mammary epithelium (Zhao & Lacasse, 2008). As a kind of polar cells, mammary epithelial cells form TJ between cells, serving as the first barrier of mammary gland tissue against outside pathogenic factors. H19 has been reported to play a significant role in maintaining intestinal barrier function by up-regulating Claudin-1 expression (Su et al., 2016). Our results demonstrated that H19 overexpression upregulated the expression of several TJ proteins of ZO-1, Occludin and Claudin-1 in MAC-T cells, which could maintain the integrity of the blood-milk barrier, thereby preventing blood components from leaking into mammary gland alveolar (Kobayashi et al., 2013). In addition, we found H19 overexpression inhibited the adhesion of *S. aureus* into MAC-T, which could protect mammary gland alveoli from further infection of pathogens.

Accumulating evidences indicate that H19 participates in a variety of inflammatory-related diseases including osteoarthritis (Steck et al., 2012), primary sclerosing cholangitis (Song et al., 2017), atherosclerosis (Han et al., 2018), ischemic neuroinflammation (Wang et al., 2017) and ulcerative colitis (Chen et al., 2016). It is well-known that LPS induces inflammatory responses by activating the NF-KB pathway (Fan et al., 2016; Ibeagha-Awemu et al., 2008). Our previous results demonstrated that H19 was involved in mammary fibrosis of dairy cows and LPS can upregulate the expression of H19 (Yang et al., 2017). In this study, we found that H19 overexpression further enhanced LPS-induced secretion of inflammatory cytokines and activation of the NF-κB pathway in MAC-T cells.

On one hand, we found that H19 was able to promote epithelial cell proliferation, β-casein secretion, TJ protein expression while inhibit *S. aureus* adhesion to cells; on the other hand, H19 could enhance LPS-induced secretion of inflammatory cytokines and activation of the NF-κB pathway in mammary epithelial cells. Similarly, H19 can play opposite roles on tumor metastasis at different stages (Matouk et al., 2016; Raveh et al., 2015). It also seems like a paradox here that H19 both represses and activates inflammation. But based on the results, we reason that H19 may perform as a part of self-recovery mechanism. When mammary tissue is exposed to proinflammatory cytokines during mastitis, highly expressed H19 introduces more intensified inflammatory response of mammary epithelial cells, resulting in a higher level of inflammatory cytokines in the mammary gland to facilitate the clearance of bacteria and their toxic substances timely, meanwhile decrease the stimulus from bacteria by repressing cell adhesion ability. During this process, the upregulation effect on TJ proteins exerted by H19 was covered up by the influence of inflammatory cytokines secretion (Mankertz et al., 2000; Xu et al., 2018). Until the end of inflammation, when the stimulus, such as LPS, was expelled or destroyed, H19 recovery effect started to show up by upregulating the expression of TJ related proteins to rebuild the blood-milk barrier.

## Conclusions

In summary, we report that H19 exerts different functions in different stage of inflammation. During inflammation, upregulation of H19 intensified the immune response of MAC-T cells by promoting the secretion of inflammatory cytokines and the activation of the NF-κB pathway to timely eliminate the pathogen. When inflammation ends, H19 overexpression upregulated the TJ proteins to restore the blood-milk barrier, which has a great significance for the recovery of mammary gland function after infection. It may provide a novel angle to understand the pathological mechanism of bovine mastitis.

##  Supplemental Information

10.7717/peerj.6715/supp-1 Table S1 Bacteria adhesion

10.7717/peerj.6715/supp-2 Table S2 Inflammatory factors were detected by ELISA

10.7717/peerj.6715/supp-3 Table S3 H19 overexpression activated inflammatory pathway

10.7717/peerj.6715/supp-4 Table S4 mRNA level of Inflammatory factors were detected by RT-qPCR

10.7717/peerj.6715/supp-5 Figure S1 H19 overexpression affected bacterial adhesion

10.7717/peerj.6715/supp-6 Figure S2 H19 overexpression weakened cell bacteria adhesion ability

10.7717/peerj.6715/supp-7 Data S1 H19 overexpression enhanced tight junction related proteins

10.7717/peerj.6715/supp-8 Data S2 Western blot-2

## Additional Information and Declarations

### Data Availability

The following information was supplied regarding data availability: The raw data are available in the Supplementary Files, which show that H19 was able to maintain normal functions and regulate immune response of bovine mammary epithelial cells.
