# Supplementary figures and images for: Overexpression of lncRNA H19 changes basic characteristics and affects immune response of bovine mammary epithelial cells
Source: PeerJ. 2019 Apr 5;7:e6715. doi: 10.7717/peerj.6715 (PMC6452850; doi:10.7717/peerj.6715)

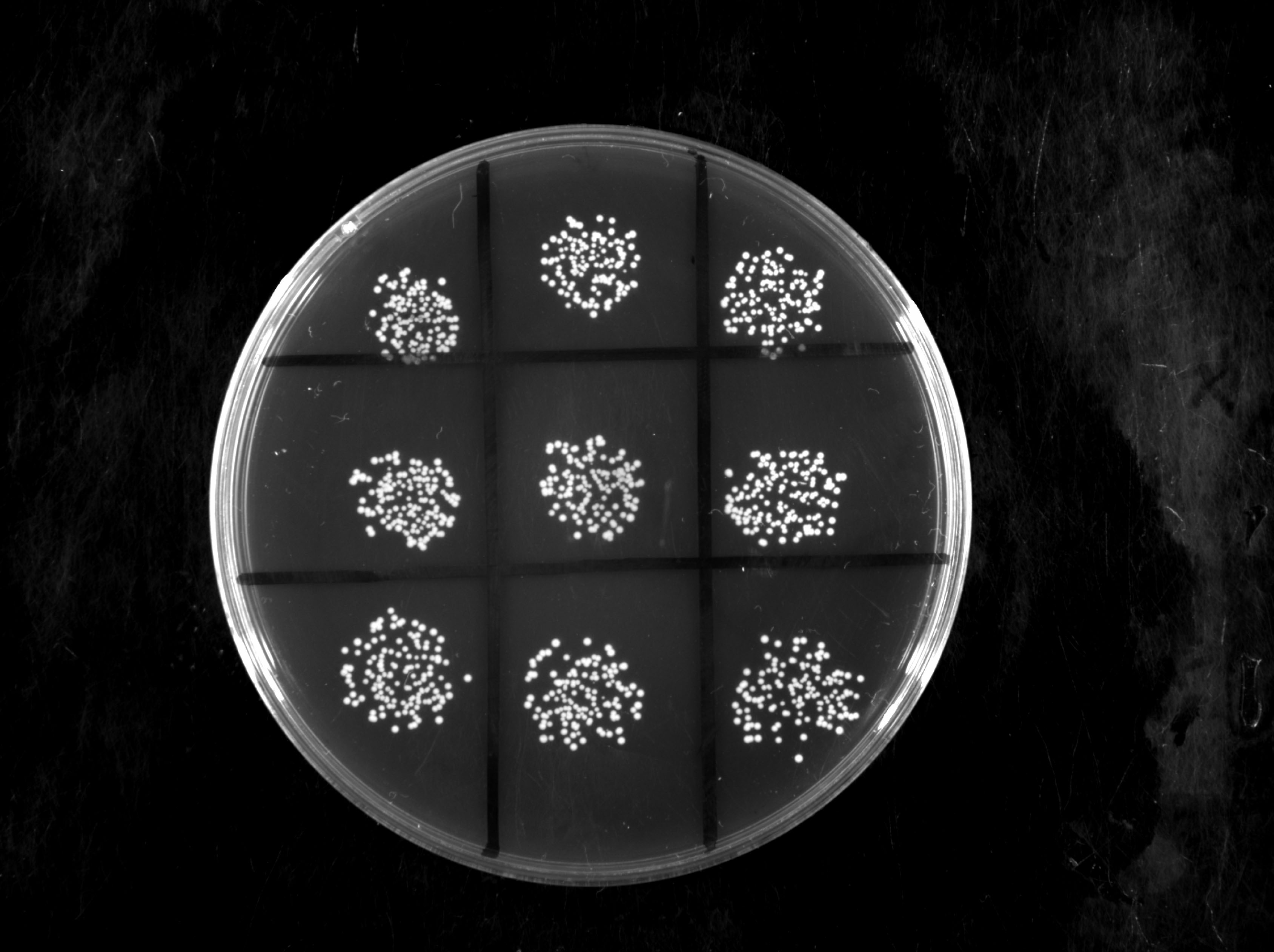

Supplement: Figure S1 [file peerj-07-6715-s005.jpg]

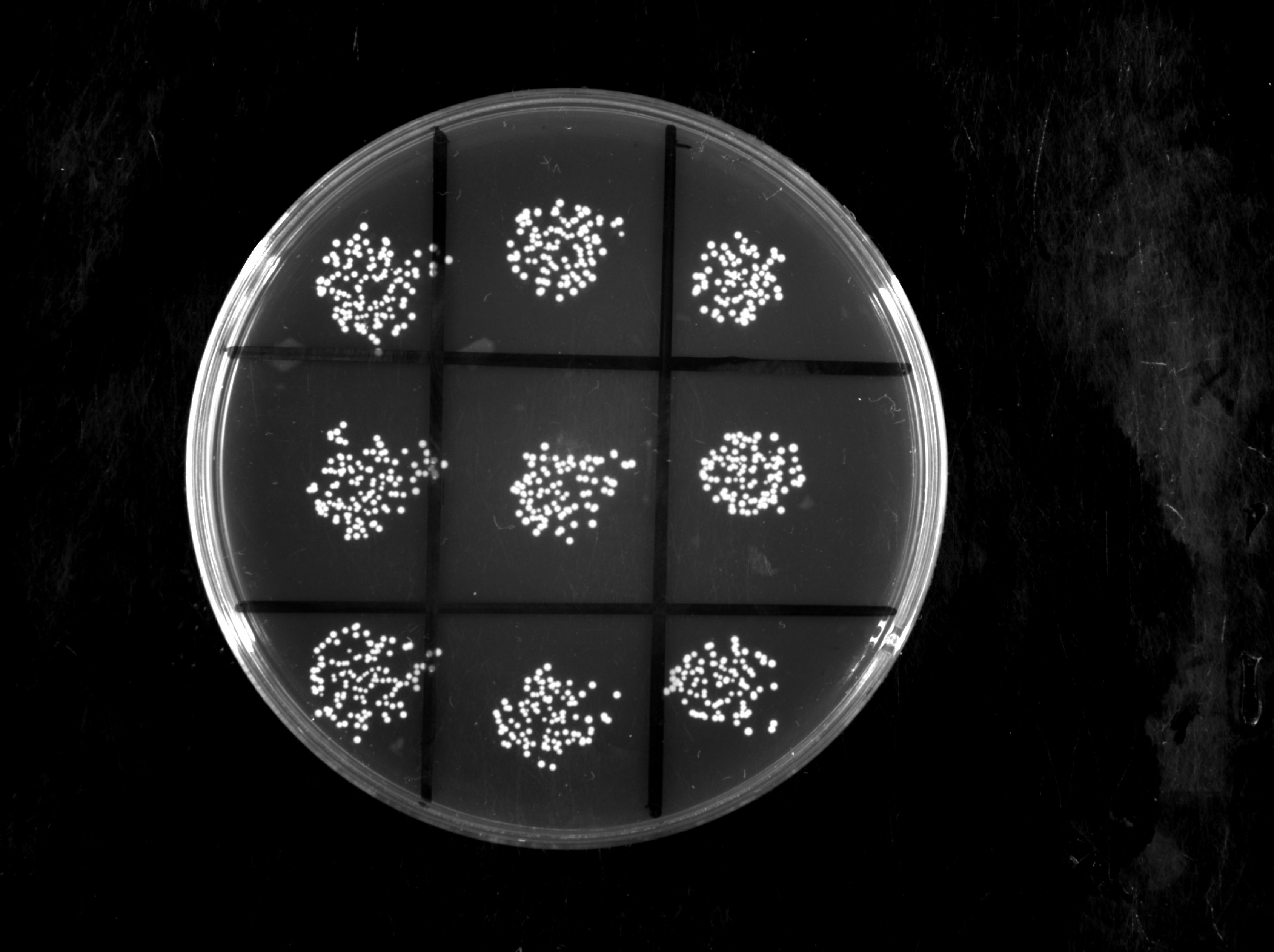

Supplement: Figure S2 [file peerj-07-6715-s006.jpg]
