# Supplementary material for: Overexpression of lncRNA H19 changes basic characteristics and affects immune response of bovine mammary epithelial cells
Source: PeerJ. 2019 Apr 5;7:e6715. doi: 10.7717/peerj.6715 (PMC6452850; doi:10.7717/peerj.6715)
Supplement: Data S1 [file peerj-07-6715-s007.zip › western blot.pptx]

## Slide 1
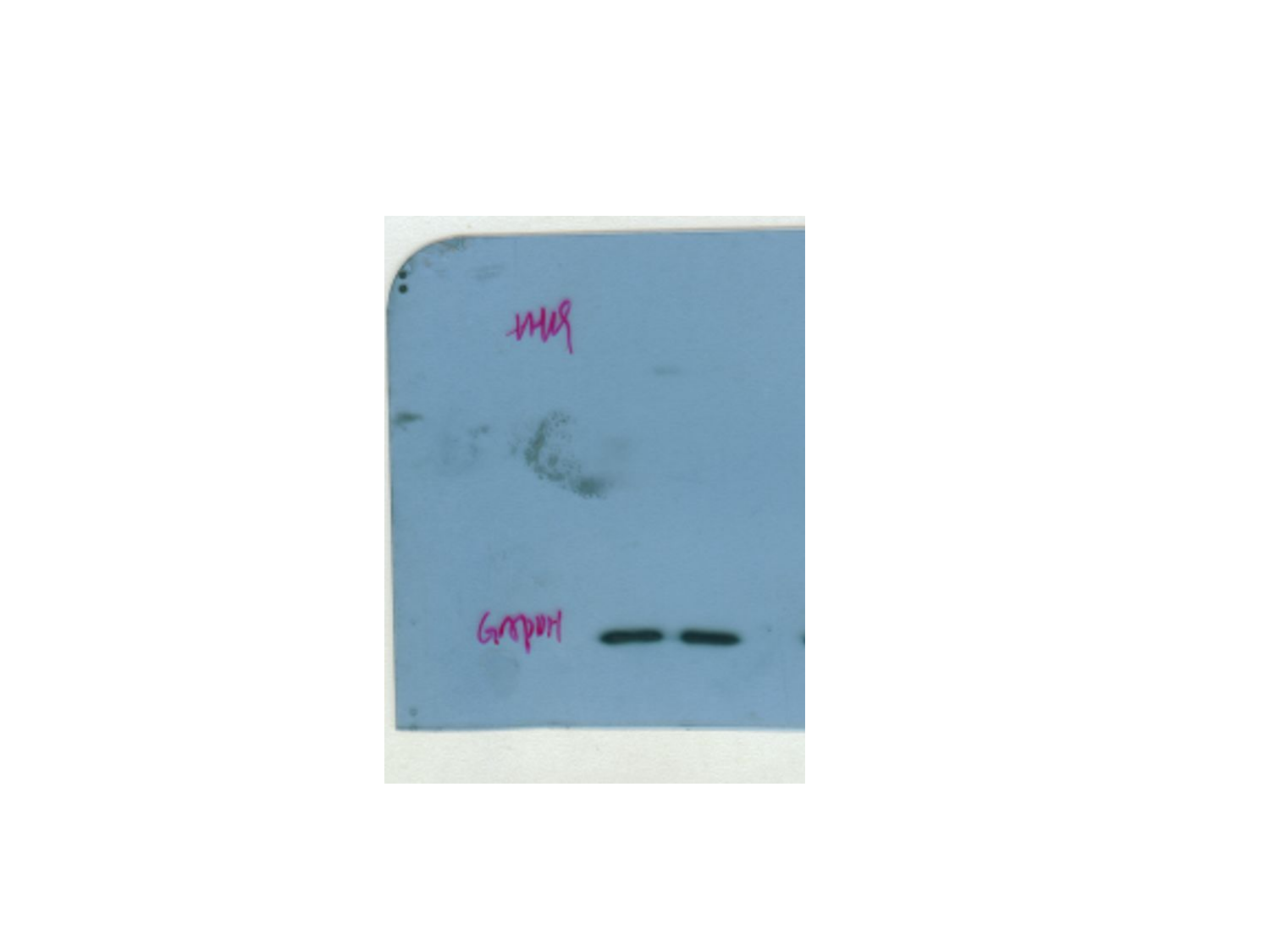

## Slide 2
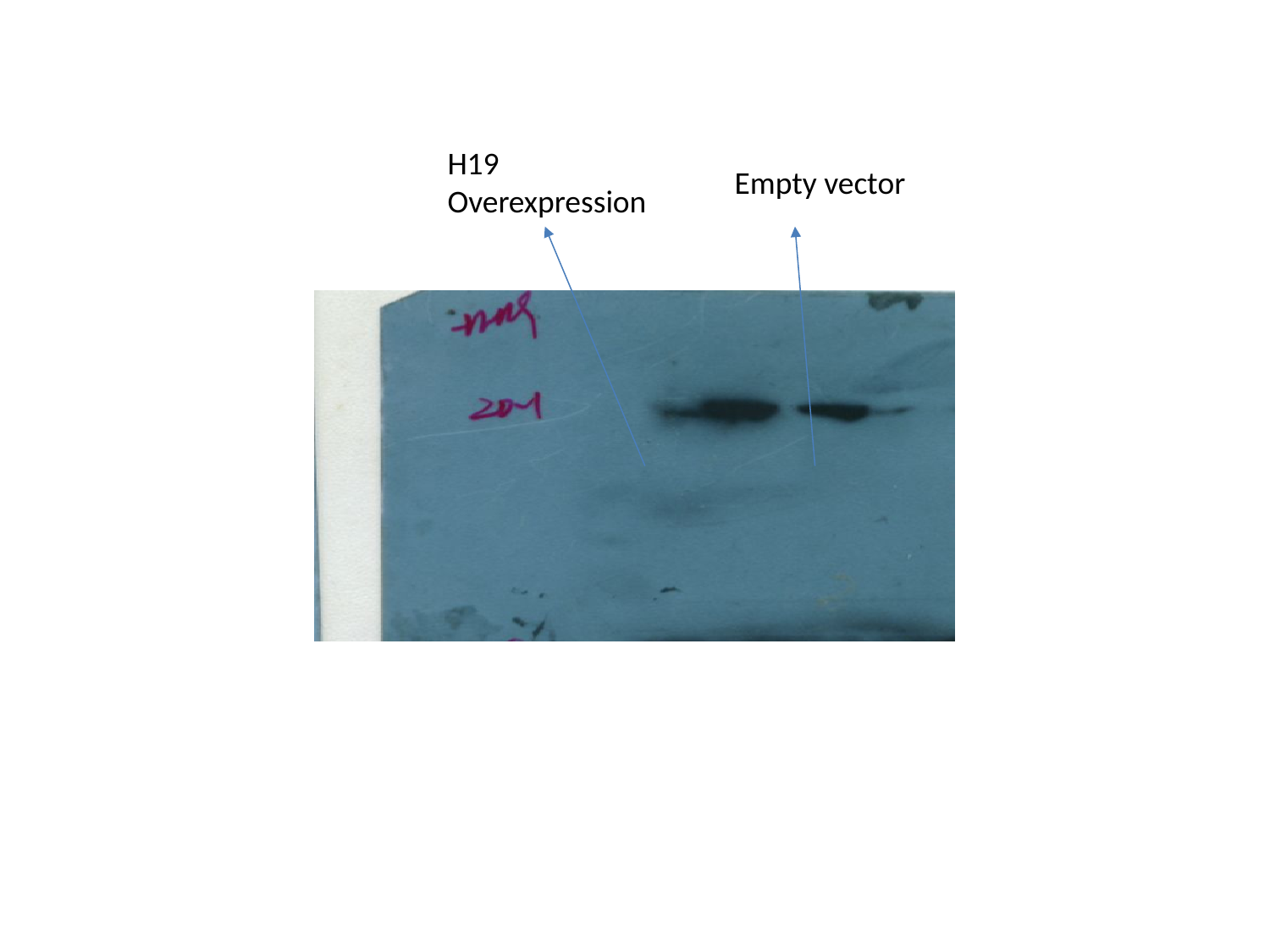

H19 Overexpression
 Empty vector

## Slide 3
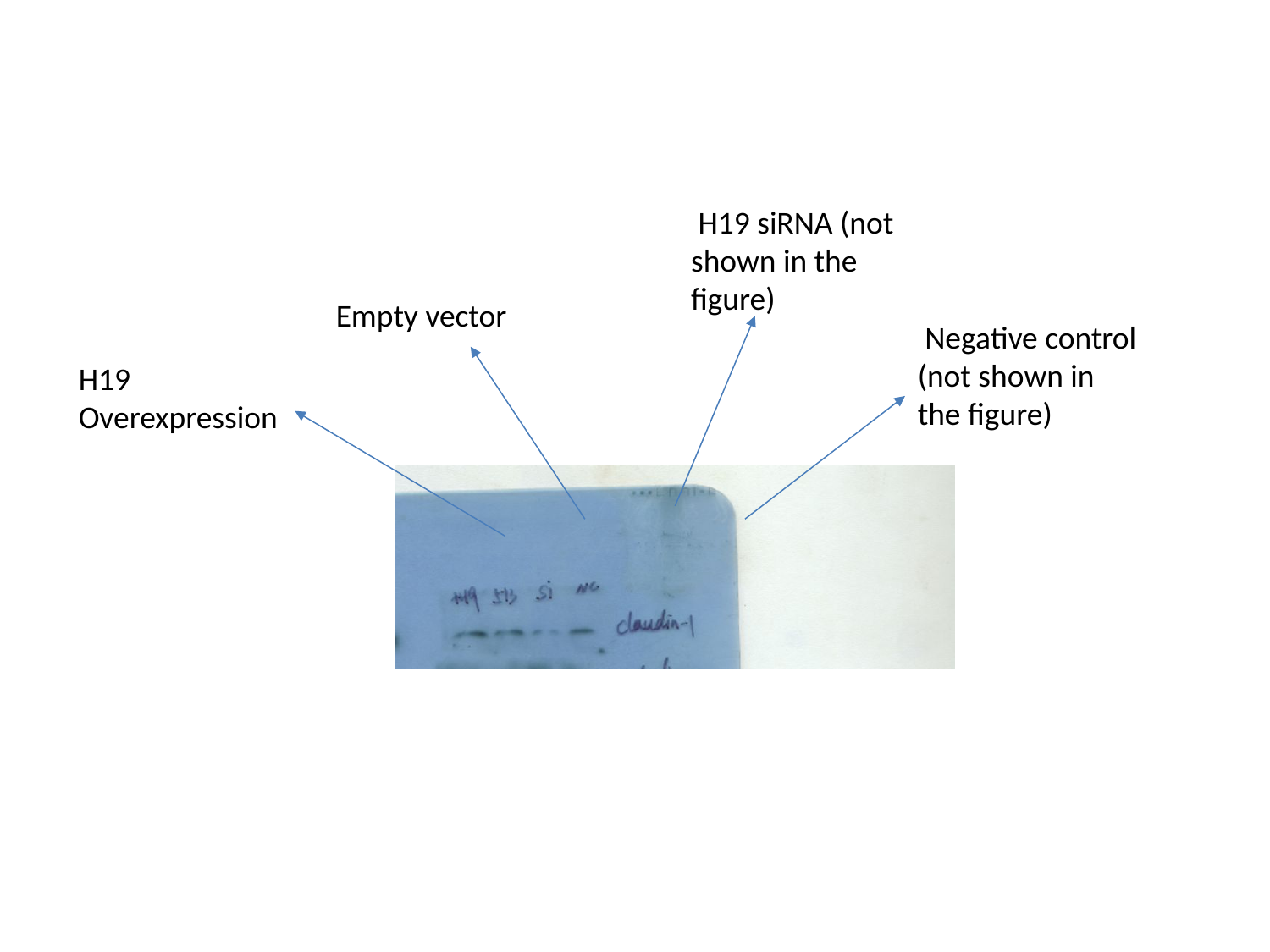

H19 siRNA (not shown in the figure)
 Empty vector
 Negative control (not shown in the figure)
H19 Overexpression

## Slide 4
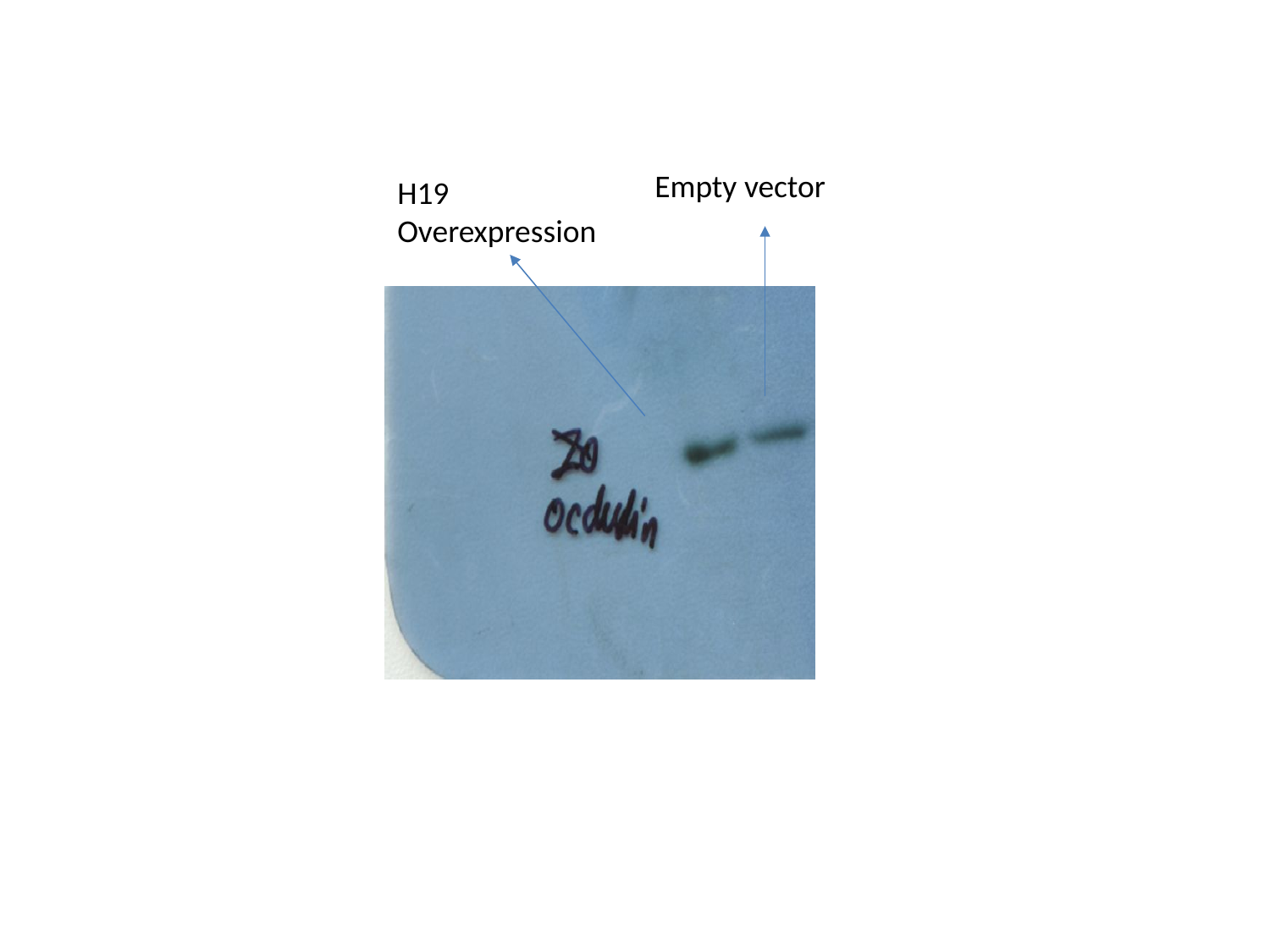

Empty vector
H19 Overexpression
