# Supplementary material for: Overexpression of lncRNA H19 changes basic characteristics and affects immune response of bovine mammary epithelial cells
Source: PeerJ. 2019 Apr 5;7:e6715. doi: 10.7717/peerj.6715 (PMC6452850; doi:10.7717/peerj.6715)
Supplement: Data S2 [file peerj-07-6715-s008.zip › WB-LXZ (2).pptx]

## Slide 1
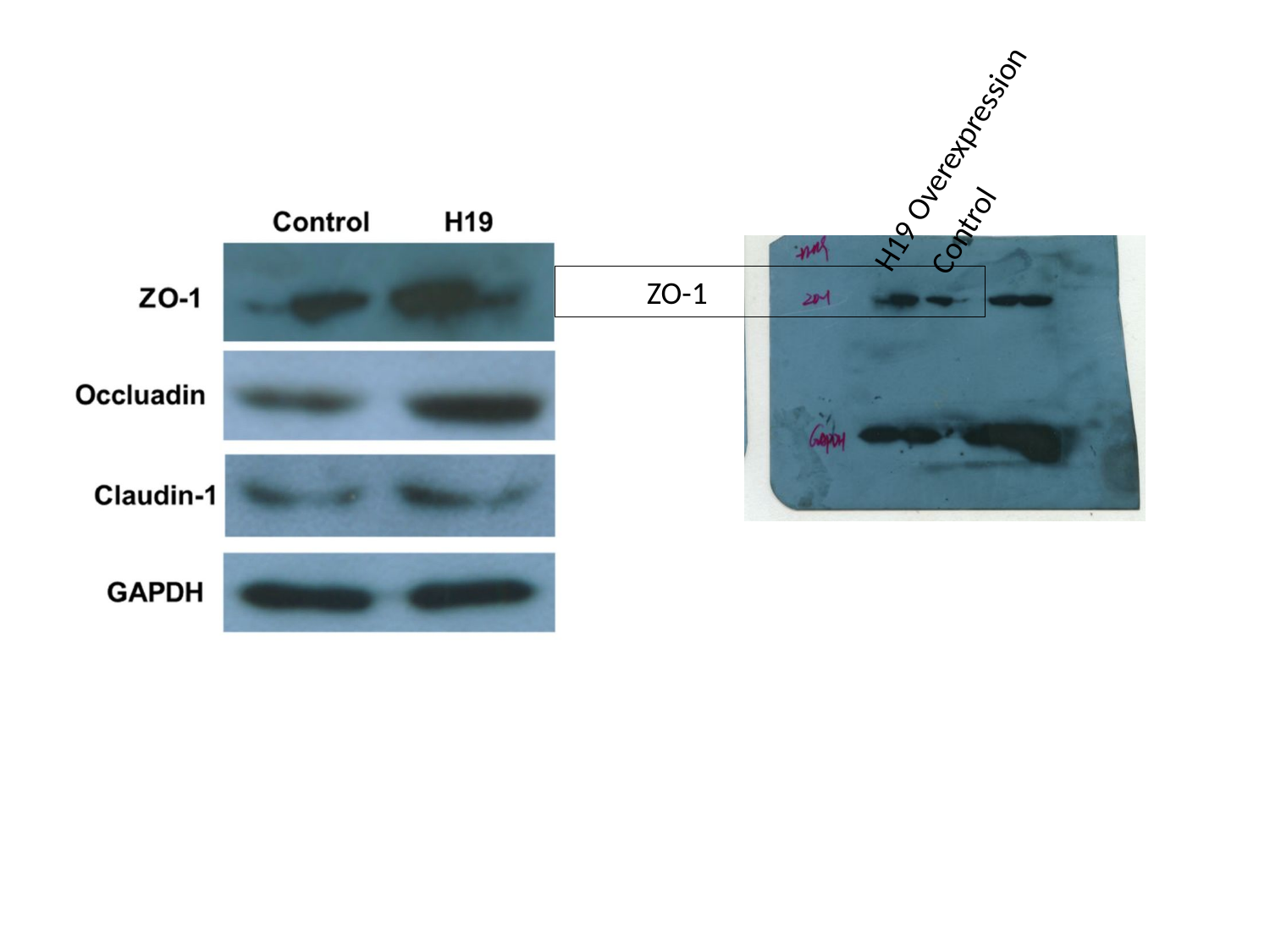

H19 Overexpression
Control
 ZO-1

## Slide 2
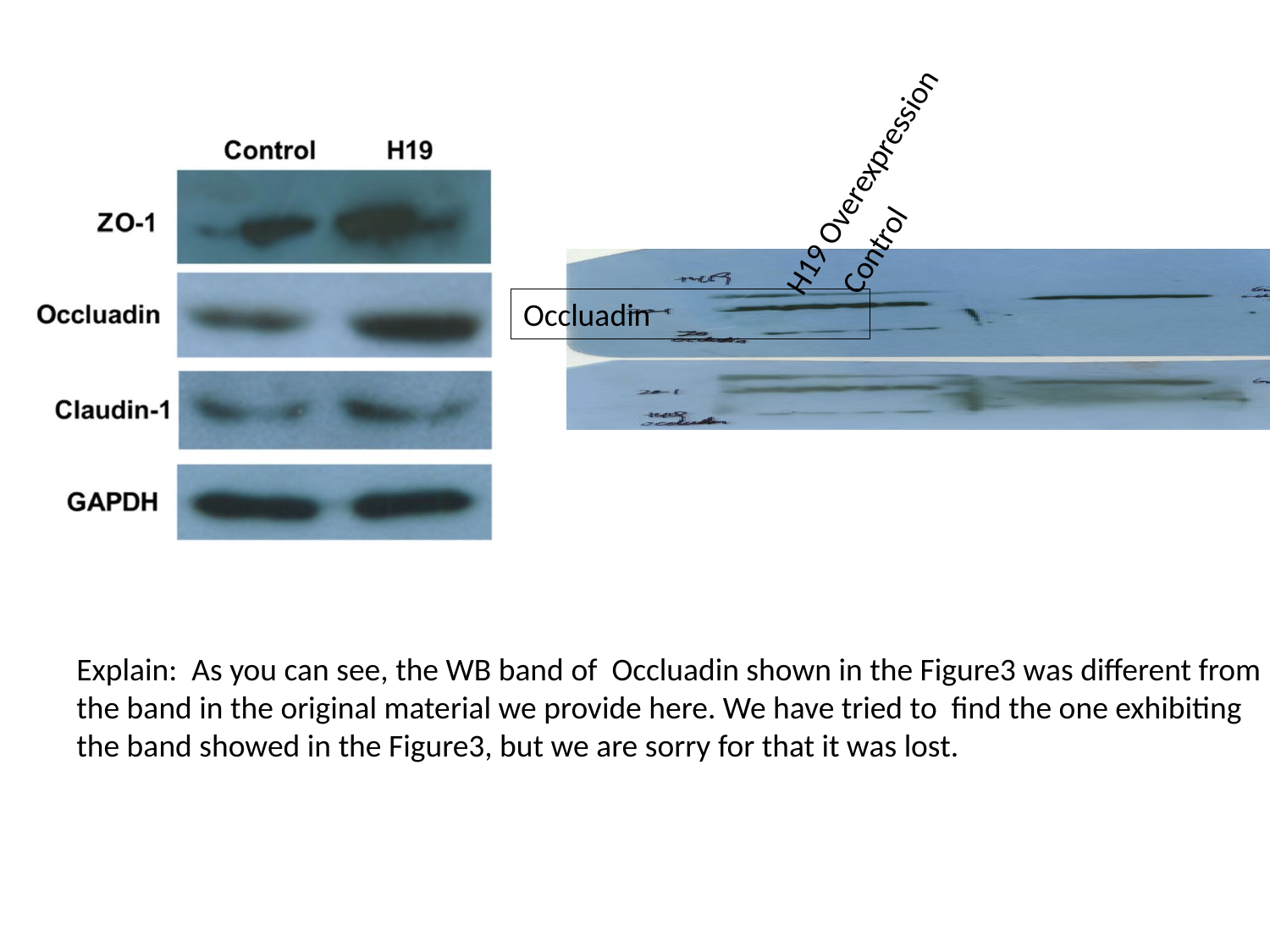

Control
H19 Overexpression
Occluadin
Explain: As you can see, the WB band of Occluadin shown in the Figure3 was different from
the band in the original material we provide here. We have tried to find the one exhibiting
the band showed in the Figure3, but we are sorry for that it was lost.

## Slide 3
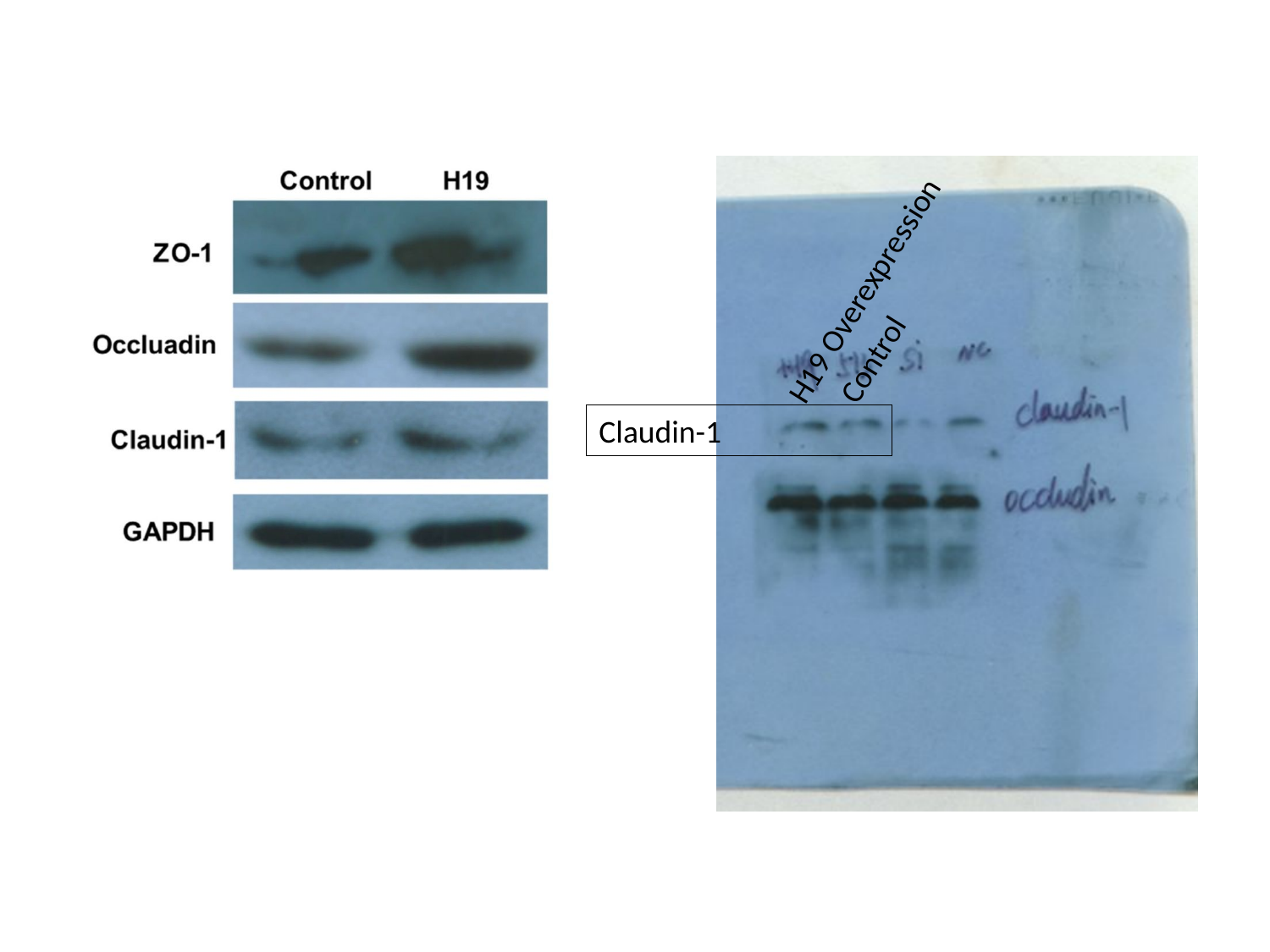

Control
H19 Overexpression
Claudin-1

## Slide 4
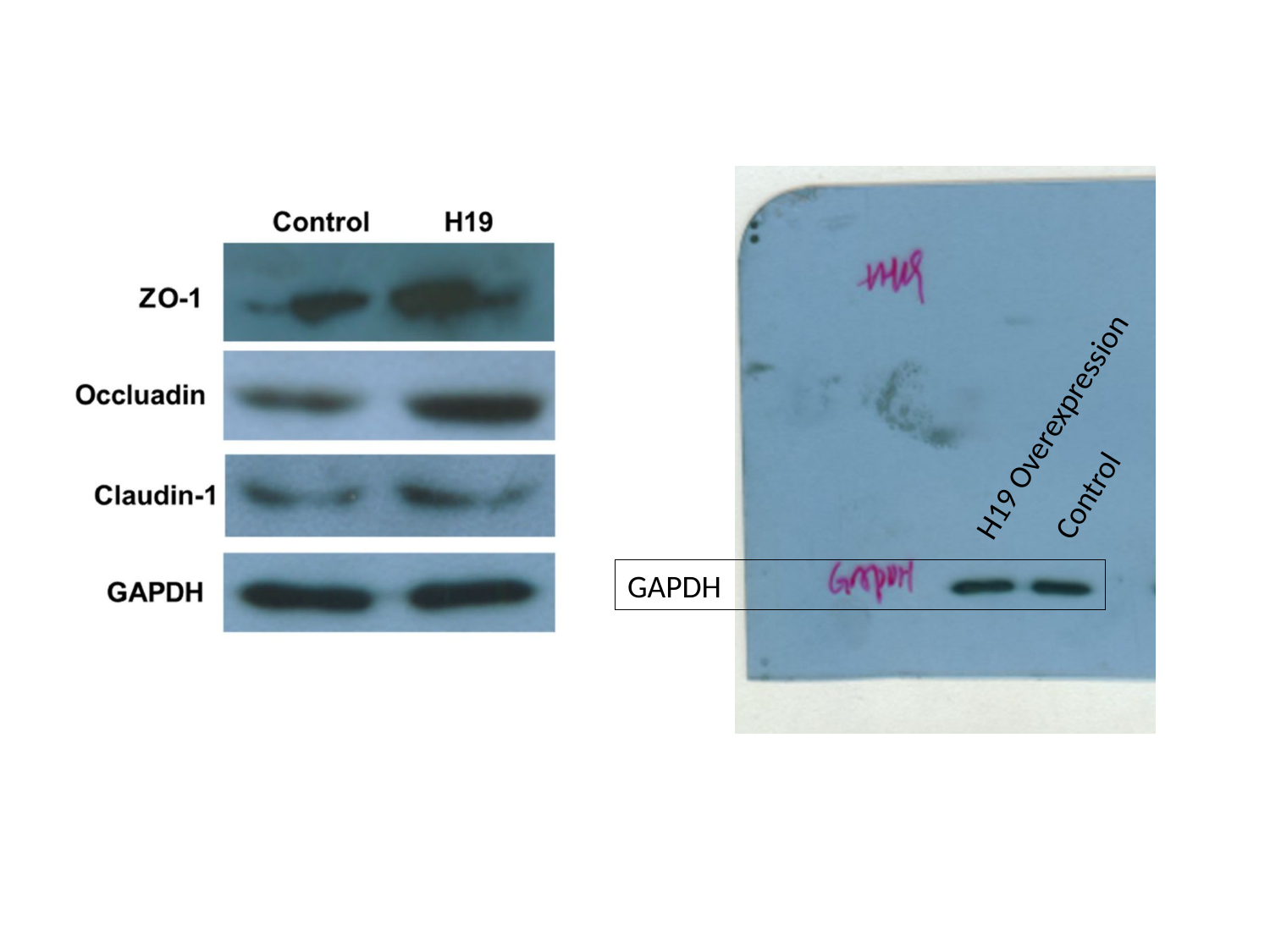

H19 Overexpression
Control
GAPDH

## Slide 5
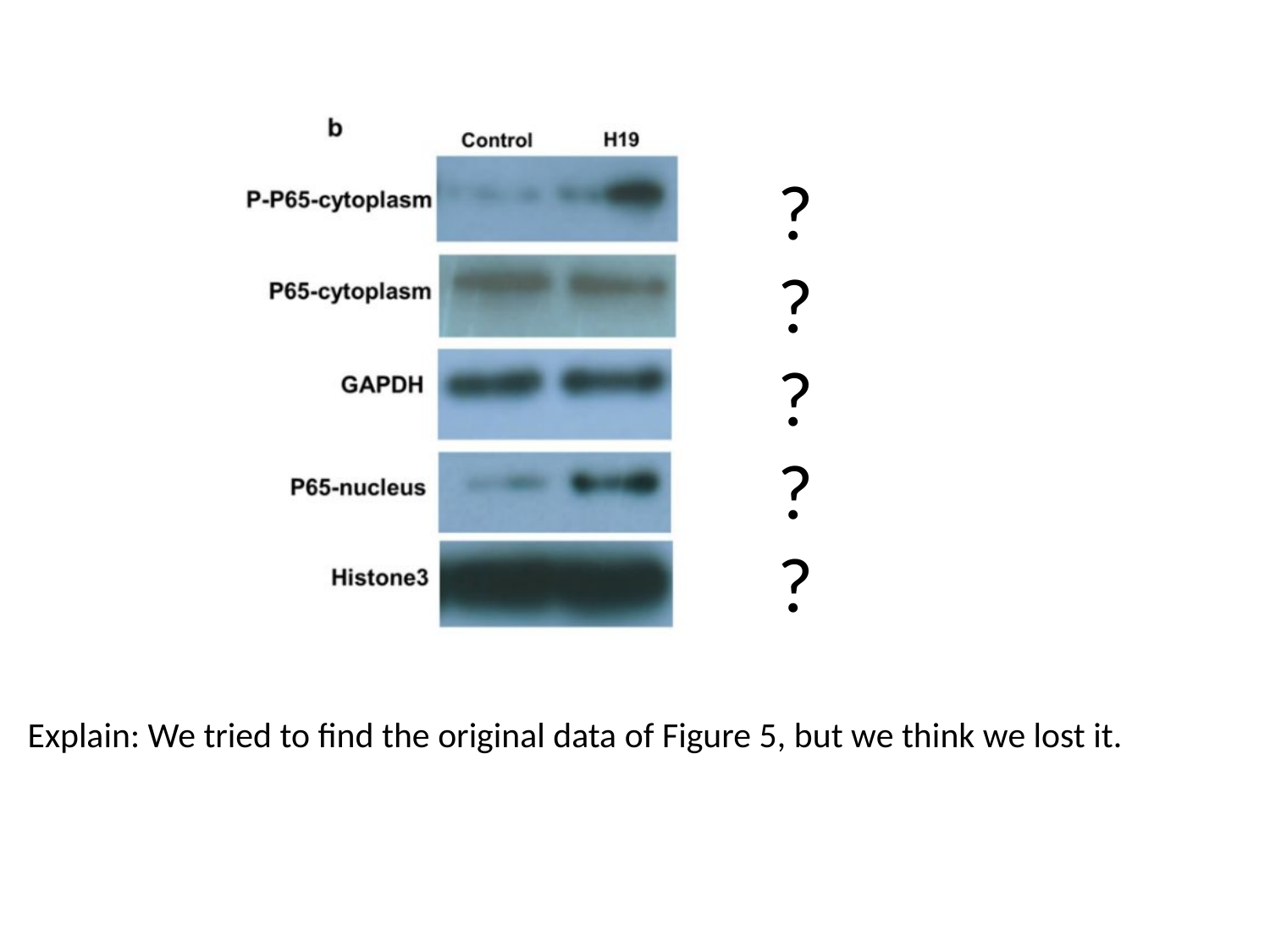

?
?
?
?
?
Explain: We tried to find the original data of Figure 5, but we think we lost it.
